# Injectable Self‐Polymerizing Hydrogel as Bone Filler for Bone Defect Treatment

**DOI:** 10.1002/mabi.202500281

**Published:** 2025-10-13

**Authors:** Xiaozhuo Wu, Jianqiu Yang, Shuai Fan, Wenbing Wan, Malcolm Xing

**Affiliations:** ^1^ Department of Mechanical Engineering University of Manitoba Winnipeg Manitoba Canada; ^2^ The Second Affiliated Hospital, Jiangxi Medical College Nanchang University Nanchang Jiangxi China; ^3^ Department of Orthopedic Surgery The Second Affiliated Hospital of Nanchang University Nanchang Jiangxi China

**Keywords:** anti‐infection, bone defect, in vivo polymerization, injectable gel

## Abstract

Poly(methylemethacrylate) (PMMA) bone cement treats bone defects that have a sol‐gel behavior that allows injection application. However, it raises concerns such as leakage, toxicity, infection, and incompatible porous structure. Here, we report an injectable gel catalyzed by silver nanoparticles (AgNPs), allowing highly efficient and biocompatible in situ polymerization to fill up the defects and eliminate infection. This gel consists of gelatin methacryloyl (GelMA) and methacrylated chitosan (ChiMA), providing a similarity to the extracellular matrix for improved cell growth, where mussel‐inspired polydopamine (PDA), reduced nano silver can be a catalyst for free radical polymerization due to its high electron activity. We further found the high surface/volume ratio of micro hydroxyapatite (HA) immobilized AgNPs for enhanced catalytic ability. The Ag‐PDA‐HA in GelMA/ChiMA produces gelation in a tunable time. After two months, the filling gel could completely eliminate *S.aureus* and raise bone volume fraction to 49.9% in infected skull models, which is approximately 30% more than contrast groups. The gel has not only increased the volume but also induced the maturation of the newly regenerated bone from H&E staining. Overall, this innovative bone filler has fast polymerization, anti‐infection, and proven bone regeneration acceleration.

## Graphical abstract

This study presents a multifunctional composite as an innovative bone cement substitute. By combining silver–hydroxyapatite–polydopamine to form a dynamic redox system that accelerates free radical polymerization, the material achieves smooth injectability. Together with methacrylated gelatin (GelMA) and methacrylated chitosan (ChiMA), the hydrogel precursor can be delivered through a thin syringe and polymerized in situ without leakage.

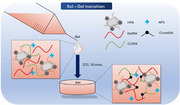

## 1 Introduction

Bone healing remains a challenge for the critical size of bone defect in modern clinical practice as it lack of natural self‐recovery ability [1, 2, 3]. Hence, treating critical bone defects often involves synthetic biomaterials for bone grafts, implants, and filler substitutes [3, 4]. Furthermore, to apply these biomaterials to bone defect site, the minimally invasive surgery (MIS) has become the preferred practice in clinics to avoid a second surgical procedure [1, 5]. Bone cement, a substitute as bone filler, is considered one of the most promising MIS methods to treat bone defects [5]. It has a setting behavior in vivo that can transit from sol‐gel, allowing MIS injection to the defect site [5, 6]. However, some traditional bone cements materials, such as poly(methylemethacrylate) (PMMA), are reported to have risks in applications that can be extended into life threatening situations [7], which include cement leakage [8, 9], loosened cement [10], heat damage to cell [5, 11], slow degradation [12], low cell viability [11], insufficient penetration into the bone microstructure [13], and potential deep infection [14].

Hydrogel‐based materials are highly favored as bone filler treatment endowed by their high‐water affinity, soft contact, resemblance to natural extracellular matrices (ECM), high biocompatibility, tailorable and flexible physical/chemical properties [15, 16, 17, 18]. Moreover, they allow injection as a liquid precursor solution and then polymerize in situ in response to various stimuli such as intermolecular interaction [15, 16], temperature [19, 20], enzymes [21], salt [18, 22], and UV light [23, 24]. This sol‐gel transition optimizes the shape matching between the implant and solid bone structure [1, 25]. There are many reports of injectable hydrogel bone fillers that have outstanding outcomes [2, 16, 26]. In our injectable bone filler, we adopted gelatin methacryloyl (GelMA) due to its high biocompatibility as gelatin is a major component of natural ECM that carries sequences to improve cell attachment and cell remodeling [27, 28, 29]. For injection application, the hydrogel precursor solution must have a viscosity that is low enough to allow being applied with the pressure of a syringe [5, 30], but also high enough to avoid spreading in vivo after injection [5, 31]. Chitosan is a prominent material that is often adopted in wound healing [32, 33]. Modification of chitosan with methacryloyl groups (ChiMA) will endow chitosan with water solubility and further cross‐linking abilities [34]. Here, we adopted ChiMA to be compounded with GelMA to tune the viscosity and maintain shape fidelity [29, 35].

To minimize in vivo leakage of bone filler after injection, the viscosity and polymerization time need to be carefully controlled [36]. Hence, it will be a plus that the polymerization time of the bone filler can be accelerated and tuned. PMMA bone cement is cured through free radical polymerization. Typically, it contains PMMA powder, methyl methacrylated (MMA) monomer solution, and benzoyl peroxide as an initiator [37]. However, both monomer residuals and the redundant free radicals caused by benzoyl peroxide are reported to be toxic to cells in the adjacent area [37]. In some applications, photoinitiation is favored due to the fast polymerization, low cytotoxicity, and highly tunable synthesis process [38, 39]. However, poor tissue penetration limits the effectiveness of this method to polymerize hydrogel precursors in vivo [39]. Hence, a green free radical system would certainly add more potential to the applications, such as injectable designs targeting deep tissues. Silver (Ag) and its salt have a wide application as catalysts in industrial applications [40, 41, 42, 43], which is relatively sparse in the biomedical area. Even the exact mechanisms in catalyzing organic reactions wait for further exploration, generally, it is considered that silver nanoparticles (AgNPs) act as an electron shuttle between reactants [42, 44]. As the main mechanism of free radical polymerization starts from electron movement from double‐bonds to form new bonds between monomers [45], therefore, we hypothesized that high electron activity induced by AgNPs can accelerate the free radical polymerization even in deep bone tissues.

To regulate the aggregation of AgNPs during synthesis, polydopamine (PDA), a mussel‐inspired polymer that enhances cell proliferation and cell adhesion [16, 46], can serve as both the capping and reducing agent to react with Ag^+^ into AgNPs [44, 47, 48, 49]. Hydroxyapatite (HA) is derived from natural bone and has excellent performance in bone restoration either alone or in combination with matrices [1] that promote the effect on osteoblast adhesion and bioactivity [50, 51, 52, 53, 54, 55]. The composition of HA's rough surface would contribute to capturing and immobilizing AgNPs to further prevent the NP aggregation, therefore, improving the catalytic efficiency.

Infection is an issue that is commonly reported derived from bone treatment [4]. To eliminate such an infection has high complexity due to how deep the bone tissues are. Ag is renowned as an antibacterial agent that has been extensively adopted for centuries [28, 56, 57]. Hence, AgNP is a plus in hydrogel precursor as both an anti‐infection agent and a catalyst for polymerization.

Joining the interests of each component, this innovative bone filler is synthesized with fulfilling the tasks of MIS injection treatment for bone regeneration and repair while avoiding the common risks. The addition of AgNPs as catalyst can promote a rapid and non‐toxic free radical propagation that enables tunable self‐polymerization of the composites without leakage. This bone cement features fast polymerization, and each component can be easily scalable to mass production with long shelf time. Moreover, the excellent anti‐infection and high biocompatibility of AgNPs could effectively and safely kill the bacteria even in the deepest tissue.

## 2 Result

### 2.1 Synthesis

**FIGURE 1:**
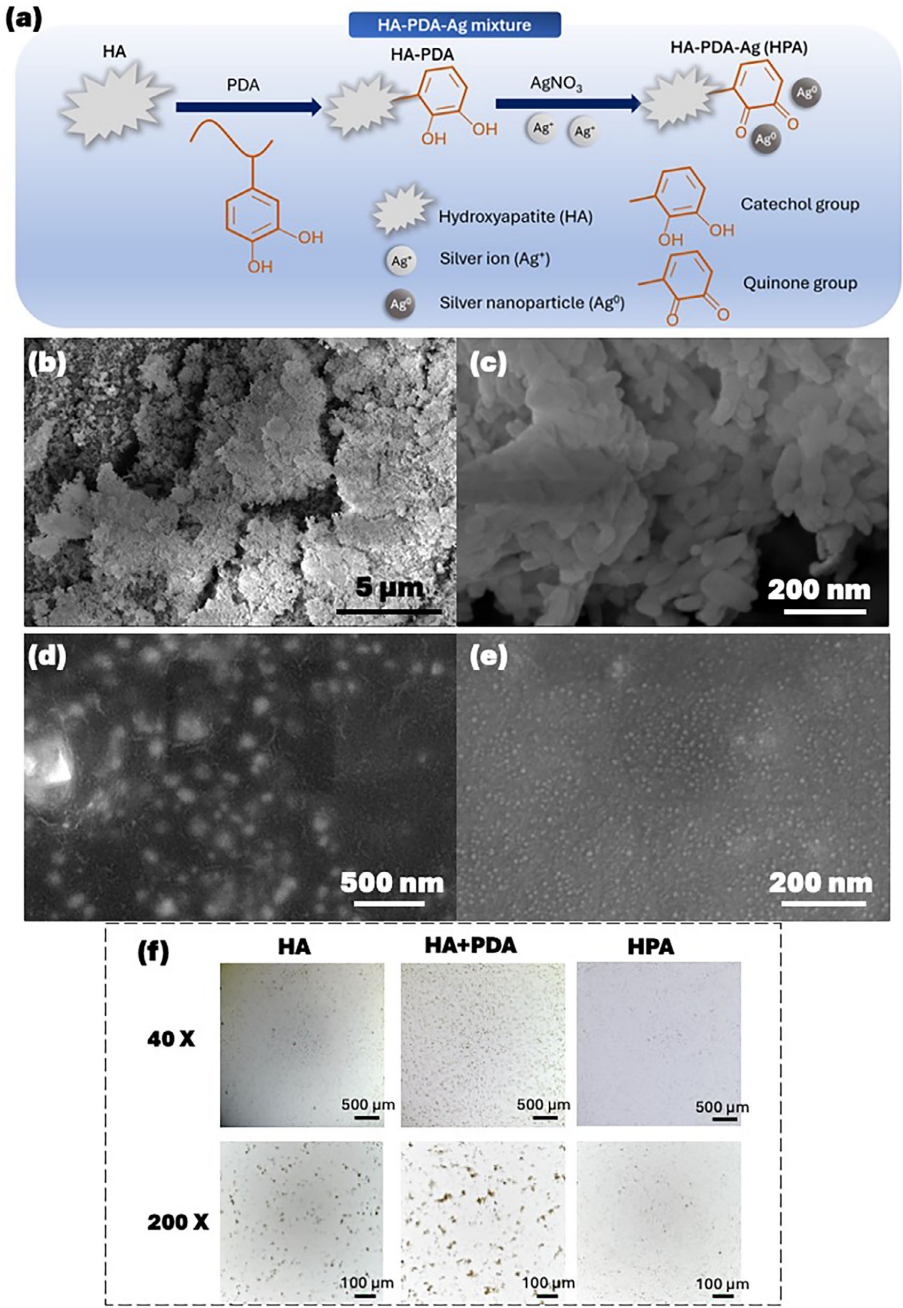
(a) Schematic demonstration of the 2‐step coating process from HA to HA‐PDA and eventually to HPA. (b) Scanning electron microscope (SEM) images of HA particles at mag. 10 000× and (c) 200 000×. (d) SEM images of AgNPs directly reduced in PDA solution at mag. 65 000×. (e) SEM images of AgNPs reduced through the 2‐step synthesis method with HA as substrate at mag. 200 000×. (f) Microscopic photos of HA, HA+PDA, and HPA powder suspended in DI water.

The HPA catalyst is a product of a facile 2‐step synthesis method. Hydroxyapatite (HA) was first coated with polydopamine (PDA) endowed with the reduction ability. Then, the mixture of HA‐PDA reduced Ag^+^ to result in the final HA‐PDA‐Ag mixture (HPA) catalyst (Figure 1a). The HA powder is an aggregation of nano‐sized particles presented as a rough surface (Figure 1b,c). Prior to using HA as a substrate to reduce Ag^+^, we reduced Ag^+^ directly in 0.2% PDA solution, resulting in AgNPs with a diameter of 103.5 ± 26.3 nm (Figure 1d). With the additional step of coating HA with PDA, the reduced AgNPs had a much smaller diameter of 10.5 ± 2.0 nm (Figure 1e). Such a small diameter of nanoparticles will result in a large surface‐volume ratio which has stronger effect on catalysis. The microscopic photos showed how the visual observations changed along with the 2‐step coating process (Figure 1f). Initially, HA powder showed a non‐uniform shape with coarse edges. Through microscopic photos, HPA had much smaller and uniformed size than HA or HA+PDA and was well dispersed. This result also matches the scanning electron microscope (SEM) inspection in Figure 1e.

**FIGURE 2:**
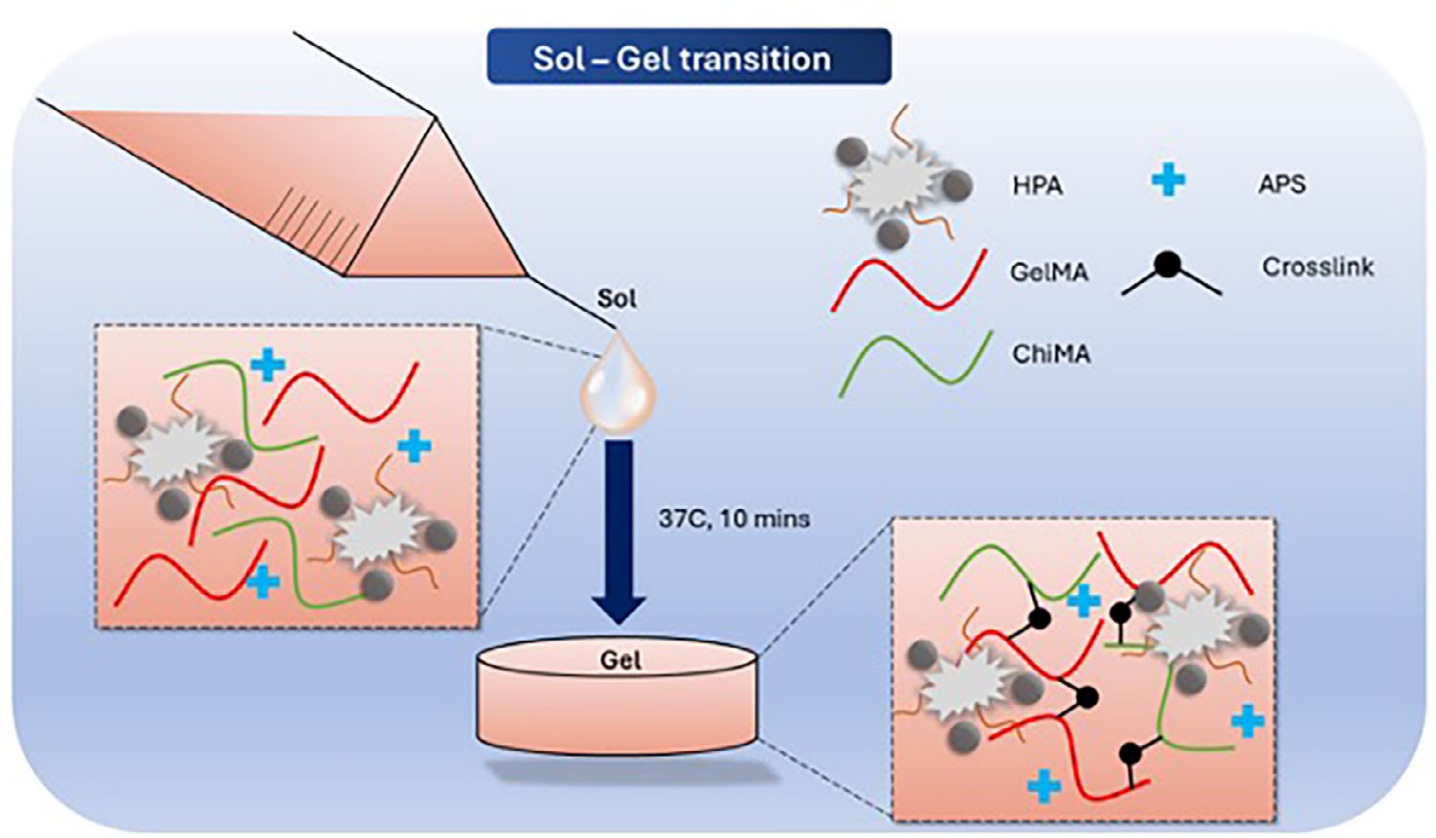
Schematic graph of the sol‐gel transition of HPA catalyzed GelMA/ChiMA dual crosslinked hydrogel.

**FIGURE 3:**
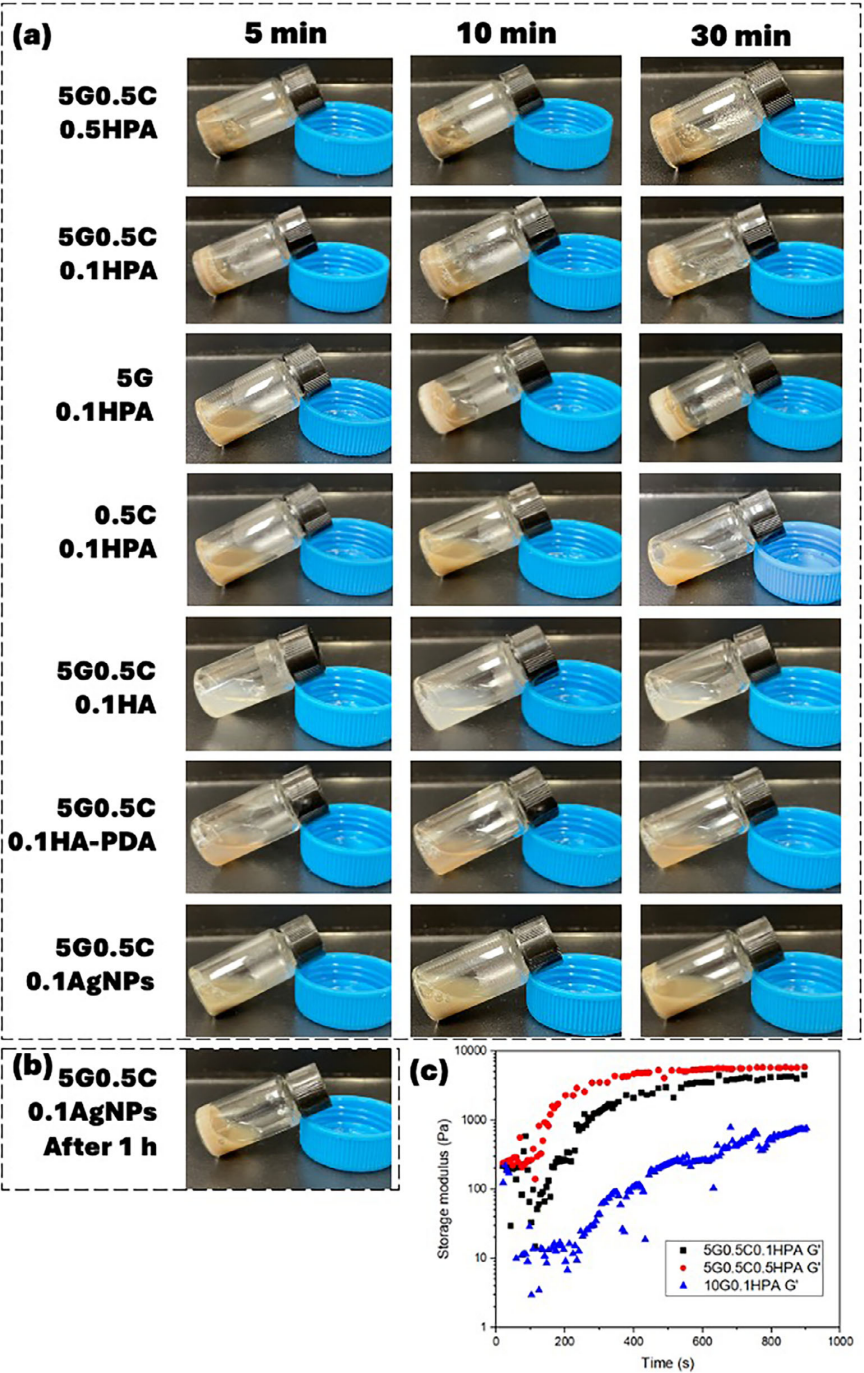
(a) Photographs of sol‐gel transition of different combinations/concentrations of G‐C‐HPA hydrogel precursor at 5, 10, and 30 min intervals. (b) 5G0.5C0.1AgNPs polymerization status after 1 h. (c) Time sweeps of G‐C‐HPA hydrogel for polymerization observation under 37°C with 1 Hz frequency.

After the 2‐step coating process, the produced HPA powder was added to the prepared GelMA and ChiMA solution to form G‐C‐HPA hydrogel precursor (Figure 2). With the presence of initiator ammonium persulfate (APS), the G‐C‐HPA hydrogel could quickly change from sol to gel within 2–10 min (Figure 3a). There is a range of polymerization times. We assume this was due to the amount of attached PDA‐Ag on the HA surface was not always uniform. From the time sweep, it could be estimated that the hydrogel started the polymerization very early, as proved by the rising storage modulus and started to reach a plateau ≈400 s signaling the completion of polymerization (Figure 3c). 0.5% HPA showed a higher elasticity response than 0.1% HPA during the polymerization. It was also noticeable that 10% GelMA without ChiMA had a continuously rising storage modulus and did not reach completion within 900 s. The storage modulus of 10% GelMA was lower than that of the G‐C dual hydrogel. This result proved the positive effect of ChiMA in the composited hydrogel precursor in that it could accelerate the polymerization and promote mechanical response.

The obtained hydrogels were rigid, non‐transparent, and in gray‐brown color originating from black AgNPs and brown PDA (Figure 3a). The higher the concentration of HPA in G‐C‐HPA hydrogel, the faster the polymerization was. Despite the concentration of HPA, they could form the rigid hydrogel within a practical time for clinical applications. G‐C dual‐hydrogel precursor was observed to have faster polymerization than GelMA, only comparing group 5G0.1HPA and 5G0.5C0.1HPA. While ChiMA could not polymerize solely, regardless of the catalyst. In comparison, either sole HA powder or HA‐PDA powder could not polymerize the hydrogel, proving that Ag and Ag only had the catalytic ability. The AgNPs in group 5G0.5C0.1AgNPs were synthesized by directly reducing AgNPs in PDA solution, which will result in a larger diameter of AgNPs. This larger AgNP group could show the signs of polymerization ≈30 min and eventually have a polymerized hydrogel after 1 h (Figure 3b). The larger AgNPs could also polymerize the hydrogel, but significantly slower than the composite HPA particles. These results suggested that the effective catalytic component was the Ag element. Ag catalyzes free radical polymerization through its high electron resonance and dynamic catechol/quinone system from PDA. First, the catechol groups on PDA reduce Ag ions into AgNPs and become quinone groups. Then the AgNPs reconvert quinone groups back to catechol groups due to their high surface plasmon resonance effect. This dynamic and reversible self‐redox system has been contributing to free radical generation, hence, to promote the polymerization [58]. The component of HA in the PDA‐Ag dynamic redox system substantially raised the catalytic ability, as expected due to the larger surface‐volume ratio.

### 2.2 Characterization

**FIGURE 4:**
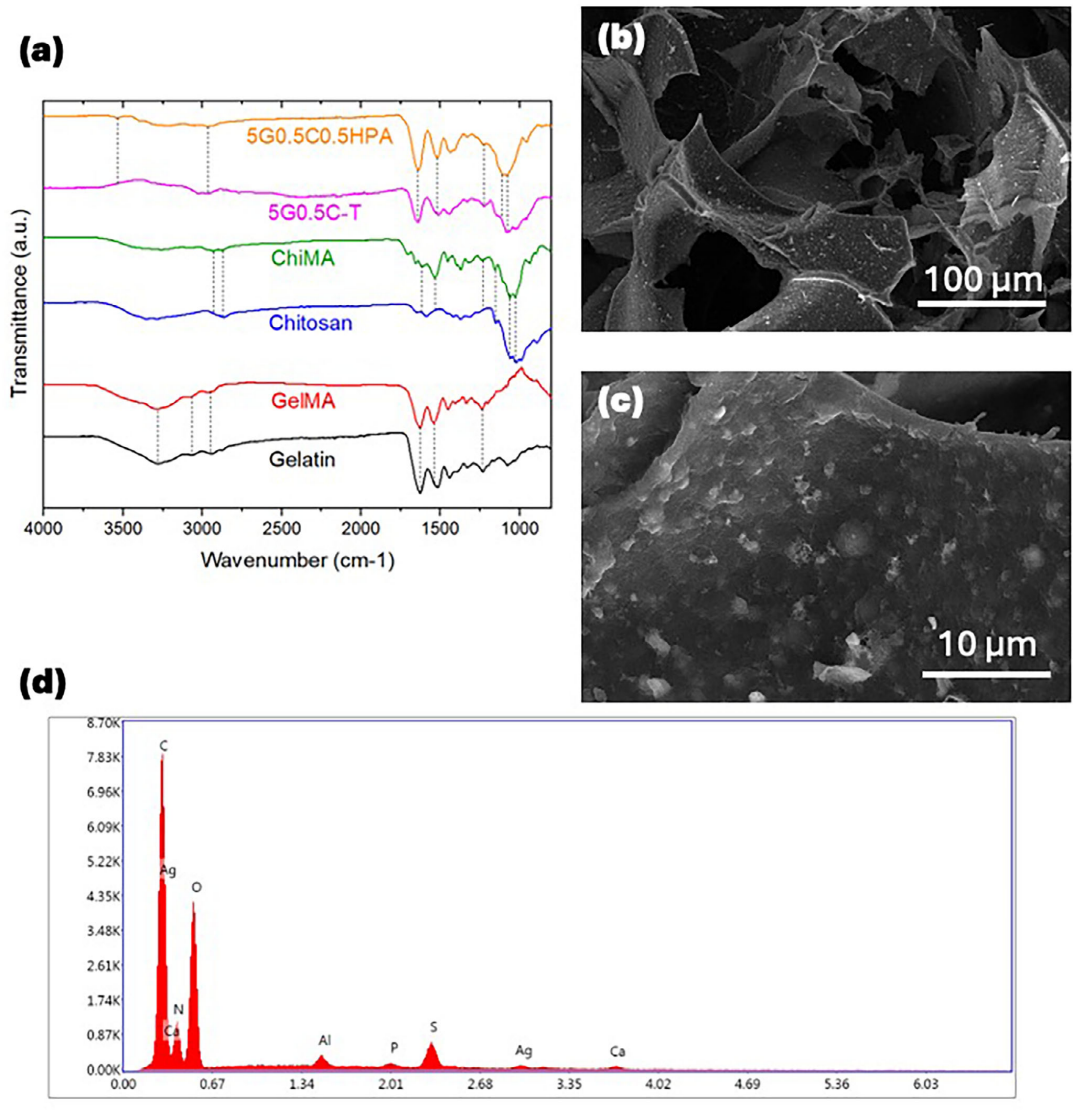
(a) The Fourier transform infrared (FTIR) spectra measurement for gelatin, GelMA, chitosan, ChiMA, 5G0.5C‐T (5% GelMA and 0.5% ChiMA catalyzed by TEMED), and 5G0.5C0.5HPA (5% GelMA, 0.5% ChiMA catalyzed by 0.5% HPA powder). (b) Scanning electron microscope (SEM) images of lyophilized 5G0.5C0.5HPA microstructure at mag. 500× (b) and 5000× (c) under SE mode. (d) Energy dispersive X‐ray spectroscopy element mapping (EDS) of sample 5G0.5C0.5HPA. It showed elements C, Ag, Ca, N, O, Al, P, S, Ag, and Ca.

The chemical structure of gelatin, GelMA, chitosan, ChiMA, and prepared hydrogels is inspected through FTIR (Figure 4a). Fourier Transform Infrared spectroscopy (FTIR) of gelatin and GelMA showed typical peaks at 1627, 1544, and 1238 cm^−1^ representing amide I, II, and III [59, 60, 61, 62]. The broad band in the range of 3200 to 3400 cm^−1^ was assigned to the peptide bond [59]. The bands at 3283 cm^−1^ were attributed to the stretching vibration of O‐H bands. The bands at 2958 cm^−1^ were attributed to the C‐H stretching vibration [59]. These absorption peaks are all in agreement with previous gelatin and GelMA studies [59, 60, 61, 62]. FTIR results of chitosan and ChiMA both showed characteristic absorption bands for chitosan at 1029 cm^−1^ for O‐H bending vibration, 1061 cm^−1^ for C‐O‐C antisymmetric stretching and 1156 cm^−1^ for O‐H stretching vibration [63]. Alkyl C‐H stretch was presented at 2873 and 2934 cm^−1^ [64]. There were three new absorption bands observed in ChiMA, which were 1620, 1535, and 1242 cm^−1^ assigned to amid I, II, and III, respectively. These new bands indicated successful methacrylation from chitosan to ChiMA [29, 64, 65, 66]. The MA grafting degree for GelMA was calculated as 85% and ChiMA was 43.3%, respectively (Figures S1 and S2).

For the composite hydrogels consisting of GelMA and ChiMA, bands assigned amides I, II, and III were at 1638, 1562, and 1238 cm^−1^. The peaks at 1078 and 1106 cm^−1^ were inherited from ChiMA, indicating the successful synthesis of a hydrogel containing both GelMA and ChiMA. The major difference between the TEMED hydrogel group and the HPA hydrogel group is that the HPA group has broad expression at 2974 to 3550 cm^−1^, indicating the O‐H vibration of PDA, while the TEMED group does not contain such characteristics [67].

The G‐C‐HPA hydrogel showed highly porous morphology with interconnected pores under SEM inspection (Figure 4b). Such porous morphology and structure of tissue engineering material are critical to new tissue and cell growth guidance, nutrient transfer, and fluid circulation [68]. The HPA appeared as nanoflakes that were well dispersed and embedded in the interconnected walls of the hydrogel matrix (Figure 4c). The presence of Ca, P, and Ag from HPA composite powder was further proved through EDS mapping (Figure 4d), suggesting the successful implementation of Ag and HA.

### 2.3 Physical Properties

**FIGURE 5:**
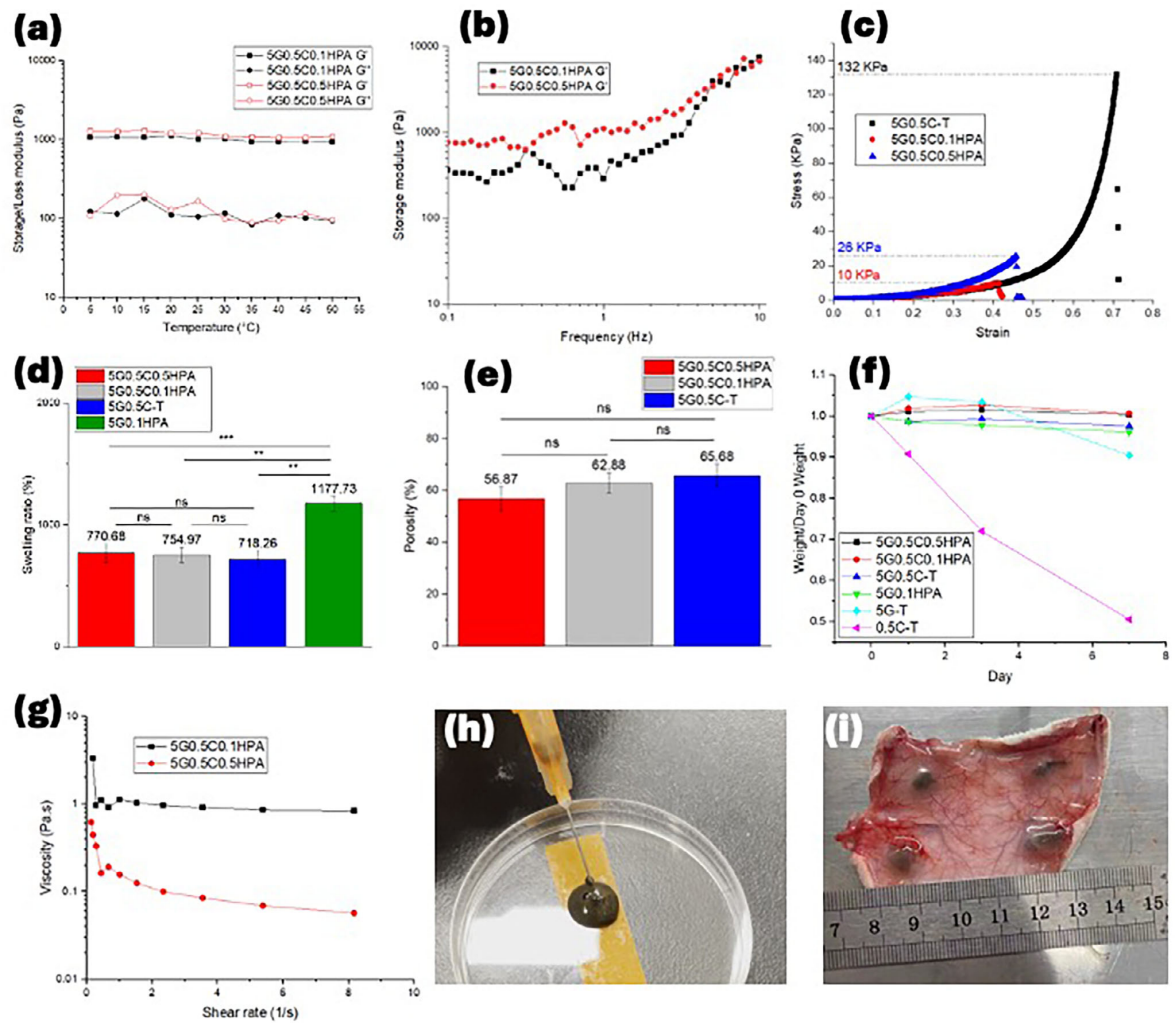
(a) Storage (*G*') and loss modulus (*G*") of G‐C‐HAP hydrogels with designed concentration changing with temperature from 5 to 50°C under a sweeping frequency of 1 Hz. (b) The storage modulus of G‐C‐HAP hydrogels was measured as a function of frequency change under 37°C. (c) Compression test of G‐C dual hydrogel catalyzed by HPA or TEMED. (d) Swelling ratio of GelMA and/or ChiMA hydrogel catalyzed by HPA or TEMED (denoted as ‐T). (e) The porosity of GelMA and ChiMA hydrogel catalyzed by HPA or TEMED (*p* < 0.05). * < 0.05, ** < 0.01, *** < 0.001. (f) Degradation of samples in PBS buffer stored in a 37°C environment. (g) Viscosity of G‐C dual hydrogel catalyzed by 0.1% HPA and 0.5% HPA. (h) 5G0.5C0.5HPA hydrogel precursor was injected through the thinnest syringe. (i) Injected 0.1% HPA precursor could polymerize efficiently under the skin of a rat without spreading within 10 min.

Upon temperature sweep of the polymerized hydrogel from 5 to 50°C, the G‐C dual hydrogel remained stable, suggesting successful free radical polymerization of GelMA (Figure 5a). In the frequency sweep, the *G*’ is dependent on the frequency, indicating the hydrogel possesses a gel property (Figure 5b). The 0.5 HPA group had a higher *G*’ value than 0.1 HPA indicating HPA content could contribute to the elasticity of the hydrogel. 5G0.5C‐T had a yield stress of 132 KPa at 61% strain of compression at break, 5G0.5C0.1HPA had a yield stress of 10 KPa at 35% strain, while 5G0.5C0.5HPA had that of 26 KPa at 46% (Figure 5c). 0.5HPA showed stronger mechanical strength than 0.1HPA as expected. The compression modulus and compression at break strain were similar to previous reports of dual GelMA and ChiMA within a typical and acceptable range [29, 69]. The contents of HA particles could increase the mechanical strength, which agrees with the literature study [29]. The Young's modulus is a parameter showing the elasticity of the material, which was determined by the initial slope coefficient of the stress/strain curve in this study. Even the 5G0.5C‐T group had a higher compression stress and tighter compression at break; its Young's modulus was calculated as the lowest of 6.50 KPa. 5G0.5C0.5HPA and 0.1HPA groups had Young's moduli of 8.66 and 8.83 KPa, respectively. Due to their complexity, the Young's modulus of natural bone and bone marrow is hard to determine and has an extensive range depending on the positions [70]. Generally, bone marrow is believed to have a Young's modulus from 0.25 to 24.7 Kpa [71]. The Young's modulus of G‐C‐HPA hydrogel falls in such a range and could make a matching physical scaffold for tissue engineering of bone marrow.

The swelling degrees of 5G0.5C0.5HPA, 5G0.5C0.1HPA, and 5G0.5C‐T were similar and were approximately sevenfolds of their original weight (Figure 5d). It suggests that the swelling ratio was not heavily affected by the catalyst but was more dependent on hydrogel composition. Sole GelMA had much higher swelling degrees, which were approximately 12 folds. Porosity test results showed no significant difference among 0.5 HPA, 0.1 HPA, and TEMED groups (Figure 5e). They were all at the range, fluctuating ≈60% of the total volume. Ideally, for MIS application, the implants should have a degradation rate matching the regeneration of the new tissues. Either too slow or too fast would either be a lack of or hinder the support of the regrowth process. However, the ideal degradation rate would not be a solid number; it should be flexible with the regeneration. G‐C‐HPA hydrogel maintained a very stable and gradual degradation rate in the first 7 days. The sole GelMA group 5G‐T had a faster drop in the hydrogel weight, and the sole ChiMA group 0.5C‐T degraded rapidly after 7 days (Figure 5f).

### 2.4 Injectability

A smooth injection is necessary for MIS applications. Both 0.1% and 0.5% HPA showed good shear‐thinning behavior that allowed the precursor to go through the syringe without much resistance (Figure 5g). Interestingly, 0.5% HPA had a lower viscosity than 0.1% HPA. We assume that this was due to the higher concentration of particles, which could hinder the formation of hydrogen bonds in the precursor. As expected, the precursor could go through the thinnest syringe, which enabled and expanded the wide application of MIS injection treatment (Figure 5h). Normally, the polymerization time must be fast to prevent the spreading of the precursor after injection. In clinical practice, it should also allow a certain time window for the operation. During experiments, we found that even though the precursor was not completely polymerized, as long as the polymerization started, the precursor would still clog the syringe. After comparison, 5G0.5C0.1HPA had the most suitable polymerization time for clinical operation (Table 1). The 0.1% HPA precursor was injected under the skin of a rat. It was observed that the precursor could easily be injected and also polymerized fast without any spreading (Figure 5i).

**TABLE 1:** Injectability of sample hydrogel precursor through standby time in vitro after adding initiator and catalyst HPA.

Concentration	1 min	2 min	5 min
5G0.5C0.5HPA	Injectable	Partially clogged due to polymerization	Completely polymerized
5G0.5C0.1HPA	Injectable	Injectable	Completely polymerized
10G0.5C0.1HPA	Injectable	Partially clogged due to polymerization	Completely polymerized
10G1C0.1HPA	Injectable but viscosity is very high	Completely polymerized	Completely polymerized

### 2.5 Cell Vitality

**FIGURE 6:**
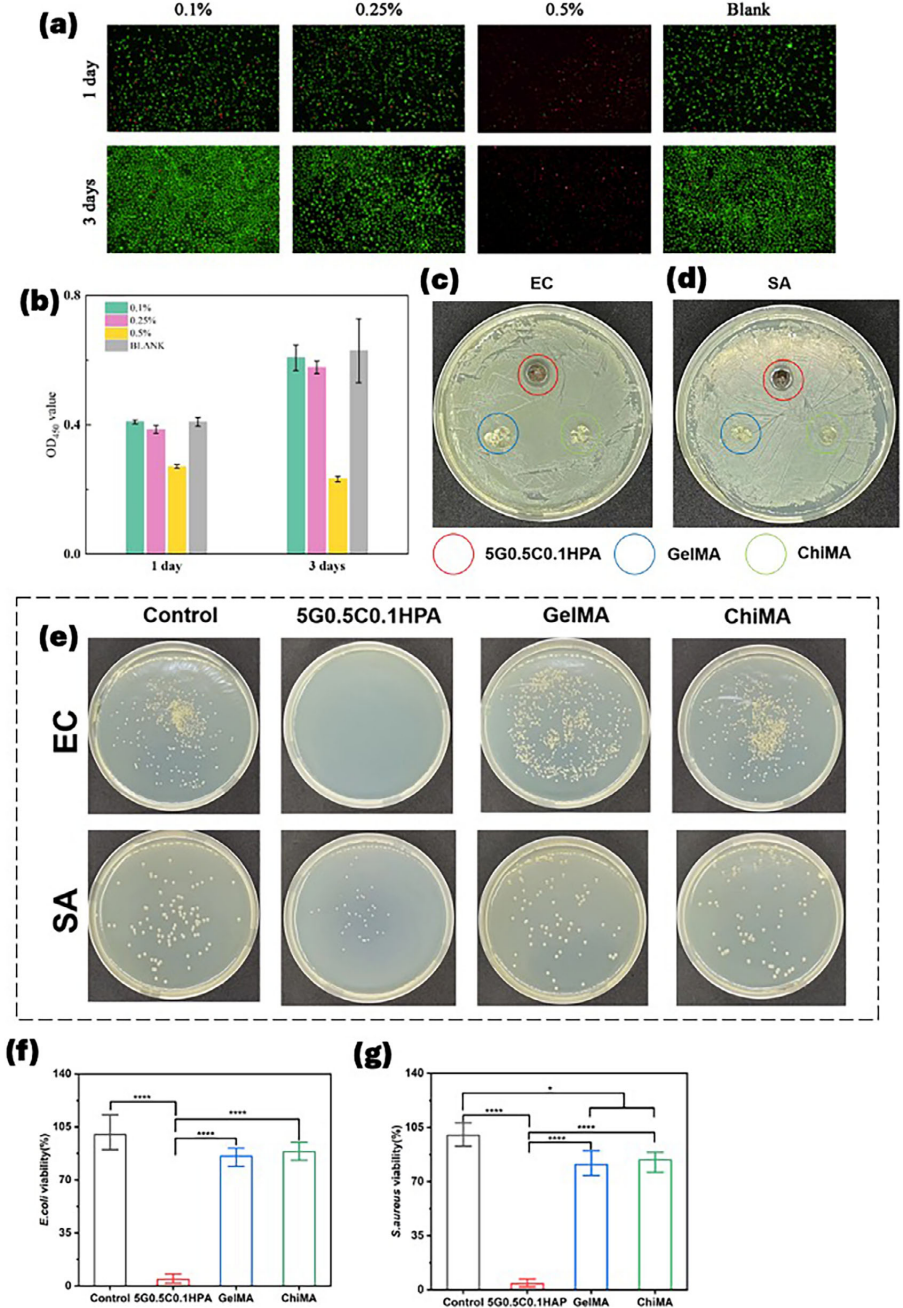
(a) Live/dead L929 cell staining cultured with different HPA concentration hydrogels of 1 and 3 days (*n* = 3). (b) Cytotoxicity of sample hydrogels with different HPA concentration hydrogels of 1 and 3 days of culture (*n* = 3). (c) Inhibitory Zones photographs produced by 5G0.5C0.1HPA, GelMA, and ChiMA cultured in E. Coli and S. aureus. (d) E. Coli and S. aureus cultured in 5G0.5C0.1HPA, GelMA, and ChiMA material. Quantification of bacterial viability for (f) *E. coli* and (g) *S. aureus. p* < 0.05 (*), *p* < 0.01 (**), *p* < 0.001 (***), *p* < 0.0001 (****).

GelMA, ChiMA, PDA, and HA are all biofriendly materials that are widely acknowledged in tissue engineering. However, the cytotoxicity of Ag elements needs to be carefully examined due to the physical and chemical nature of AgNPs that could be dependent on the concentration and size [72]. The cytotoxicity of G‐C‐HPA hydrogel was examined through L929 cell culture. The examination was grouped by different HPA concentrations. Visually, the stained images showed live/dead L929 cells, where green represents live cells and red represents dead cells (Figure 6a). The 0.1% concentration of HPA was compatible with cells that did not have a significant amount of cell death. The intensity of live cells was also like the blank group. With the increase in HPA concentration, the number of live cells dropped. At 0.5%, there were almost no living cells observed. The CCK8 evaluation also showed that 0.1% HPA had a matching cell viability with the blank group. 0.25% HPA had a lower cell viability level, while 0.5% HPA showed noticeable cytotoxicity (Figure 6b).

This result proved that the 5G0.5C0.1HPA was biofriendly and caused no harm to cells. The higher cytotoxicity observed in the 0.5HPA group was attributed to the increased concentration of Ag. Therefore, the Ag content must be carefully controlled to balance antibacterial efficacy with cytotoxic risk. The AgNP content in 1 mL of the 5G0.5C0.1HPA sample (equivalent to 1 mg of HPA powder) was approximately 353 µg (Table S1). Within the polymerized hydrogel, AgNPs were immobilized on the surface of HA and further encapsulated within the hydrogel network. Previous studies have discussed how cytotoxicity depends on factors such as particle size, concentration, and site of application [73]. However, there is no universally accepted threshold for safe Ag concentrations across all biomedical applications. In general, lower concentrations are required for applications involving oral or inhalation exposure, while higher levels are tolerated in localized uses such as bone implants [74, 75, 76]. Compared to previously reported silver concentrations in bone cements [77, 78], our sample with approximately 0.035 wt.% Ag, is considered to be within a safe range.

### 2.6 Anti‐Bacteria

One of the most important usages of Ag is its well‐known bacterial killing ability through contact with the cell walls of bacteria [28, 56, 57]. We expected the HPA catalyst coated with Ag would have positive results in anti‐bacteria. The inhibitory zone test of sliced hydrogel samples showed the 5G0.5C0.1HPA sample could effectively kill *E. coli* and *S. aureus* bacteria (Figure 6c,d). GelMA and ChiMA hydrogel did not kill the bacteria around the placed samples. Compared to blank control groups, 5G0.5C0.1HPA sample eliminated all *E. coli* with no visible bacteria left (Figure 6e). It demonstrated strong antibacterial activity against *S. aureus*, effectively eliminating a large number of bacteria. In contrast, GelMA and ChiMA showed minimal antibacterial effects, with little evidence of bacterial elimination (Figure 6f,g). The anti‐bacterial study further proved that the method of coating Ag onto HA does not only act as a catalyst but is critical in tissue engineering material to eliminate bacteria. Even only 0.1% of HPA could have such a strong anti‐bacterial effect, primarily due to the sustained release of Ag ions [79]. Over time, AgNPs gradually oxidize into Ag⁺ ions and are released (Figure S3). After 24 h in water, the 0.1HPA group released Ag⁺ ions at a concentration of 126.90 ± 18.43 µg mL^−1^. This concentration significantly declined over time, reaching 22.62 ± 4.63 µg mL^−1^ on day 3, 4.38 ± 1.69 µg mL^−1^ on day 5, and 0.54 ± 0.28 µg mL^−1^ on day 7. This sustained Ag ion release proved the effectiveness of the catechol/quinone dynamic redox system that is not only in catalyzing the initial release but also in maintaining continuous Ag⁺ ion generation throughout the application period.

### 2.7 In Vivo Skull Defect Healing

The bone defects were created on female rats’ skulls to study the in vivo performance of the G‐C‐HPA injectable hydrogel. To maximize the potential of the hydrogel, the defect was applied with *S. aureus (SA)* bacteria to monitor the hydrogel performance in an infection model. The defects were created by electric drill on rats’ skull with a diameter of 5 mm. After the infection, the defect was dressed in pre‐made GelMA as a contrast group. The concentration of 5G0.5C0.1HPA was selected for in vivo study judging by the high biocompatibility, outstanding antibacterial activity and smooth injection. The 5G0.5C0.1HPA hydrogel precursor was injected into the defects and subjected to self‐polymerization afterwards. It was observed that the hydrogel precursor could polymerize within 10 min on all rats. The defect wounds were then sutured for healing.

**FIGURE 7:**
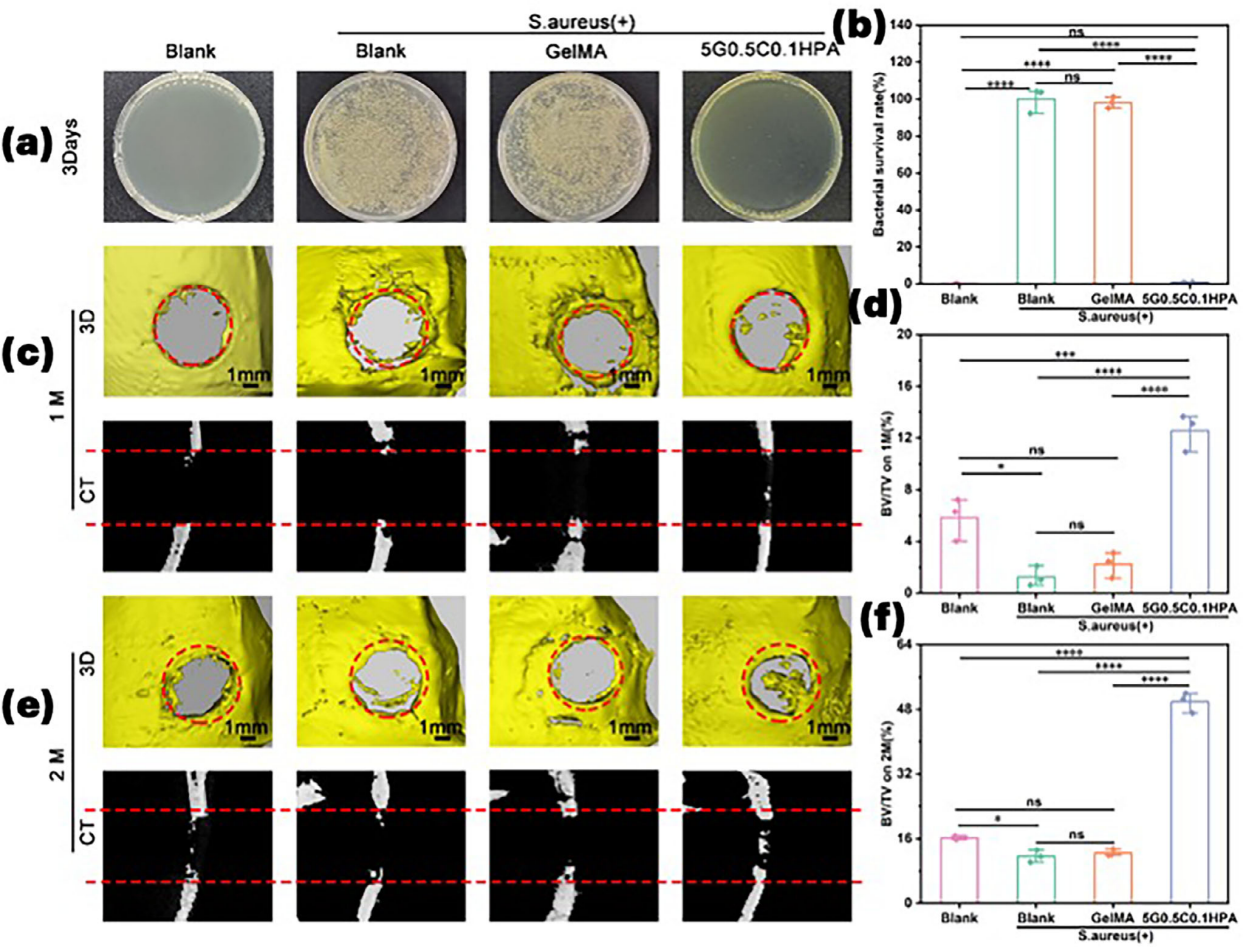
(a) Photographs of in vivo bacteria development after 3 days cultured on the skull defect. (b) Count of the bacterial survival rate cultured from the skull defect. (c) Photographs and CT scan of the bone healing process after 1 month. (d) Measured bone volume fraction after 1 month of treatment. (BV: bone volume; TV: tissue volume). (e) Photographs and CT scan images of the bone healing process after 2 months. (f) Measured bone volume fraction after 1 month of treatment. *p* < 0.05 (*), *p* < 0.01 (**), *p* < 0.001 (***), *p* < 0.0001 (****).

The bacterial infection on wounds could be fatal for patients, especially when it happens on an internal bone defect as it is harder to reach. After 3 days of the in vivo defect was made, the antibacterial activity was evaluated through washing *SA* from the bone marrow cavity (Figure 7a). Visually, *SA* blank group and *SA* GelMA groups had a growing amount of *SA* bacteria. *SA* GelMA had 100.01 ± 3.83 % bacterial survival rate, and GelMA group had 98.13 ± 1.72 % bacterial survival rate (Figure 7b). This shows that even GelMA is acknowledged as an excellent tissue engineering material, it has no in vivo antibacterial ability. In contrast, negative blank group had no visual bacteria with only 0.08 ± 0.04 % survival rate. 5G0.5C0.1HPA sample group showed minimal visual bacteria in the medium with only 0.72 ± 0.17 % survival rate. Such excellent antibacterial ability was a result of 0.1% of HPA component, which was coated with AgNPs. With such a low concentration of 0.1%, the HPA component could effectively inhibit the bacteria in vivo, also maintains a high biocompatibility.

After 1 month of treatment, the photographs were taken and shown in Figure 7c. The negative blank group had minimal changes, with a healing bone volume fraction (BV/TV) of 5.85 ± 0.95 %. The infected *SA* blank and *SA* GelMA group both had developed a rough edge along the defect, indicating the healthy skull bone tissue adjacent to the man‐made defect was infected and corrupted. The healing performance of these 2 groups was worse than the negative blank group caused by the infection. Their BV/TV were 1.25 ± 0.44 % (SA blank) and 2.25 ± 0.57 % (SA GelMA) (Figure 7d). *SA* 5G0.5C0.1HPA group had the best healing process among all. First, it is noticed that the edge of the defect remained smooth, showing the defect was not affected by the bacteria infection. In CT scan, the bone tissue also started its growth within the defect area, leading to a higher BV/TV of 12.56 ± 0.85 %.

After 2 months of treatment, the difference between the hydrogel and contrast groups was even more pronounced (Figure 7e). The negative blank group was left to natural healing with no treatment or infection. After 2‐month its BV/TV eventually reached 16.21 ± 0.27 %. *SA* blank and *SA* GelMA groups had overcome the corruption but still left some noticeable marks along the defect circle shown in the CT scan. In comparison, our 5G0.5C0.1HPA promoted the healing of the skull bone tissue with a high BV/TV ratio of 49.90 ± 1.44% (Figure 7f). No sign of infection or corruption was observed either. Comparing to natural healing, it is proved that G‐C‐HPA composite hydrogel can significantly and safely accelerate the bone healing process.

**FIGURE 8:**
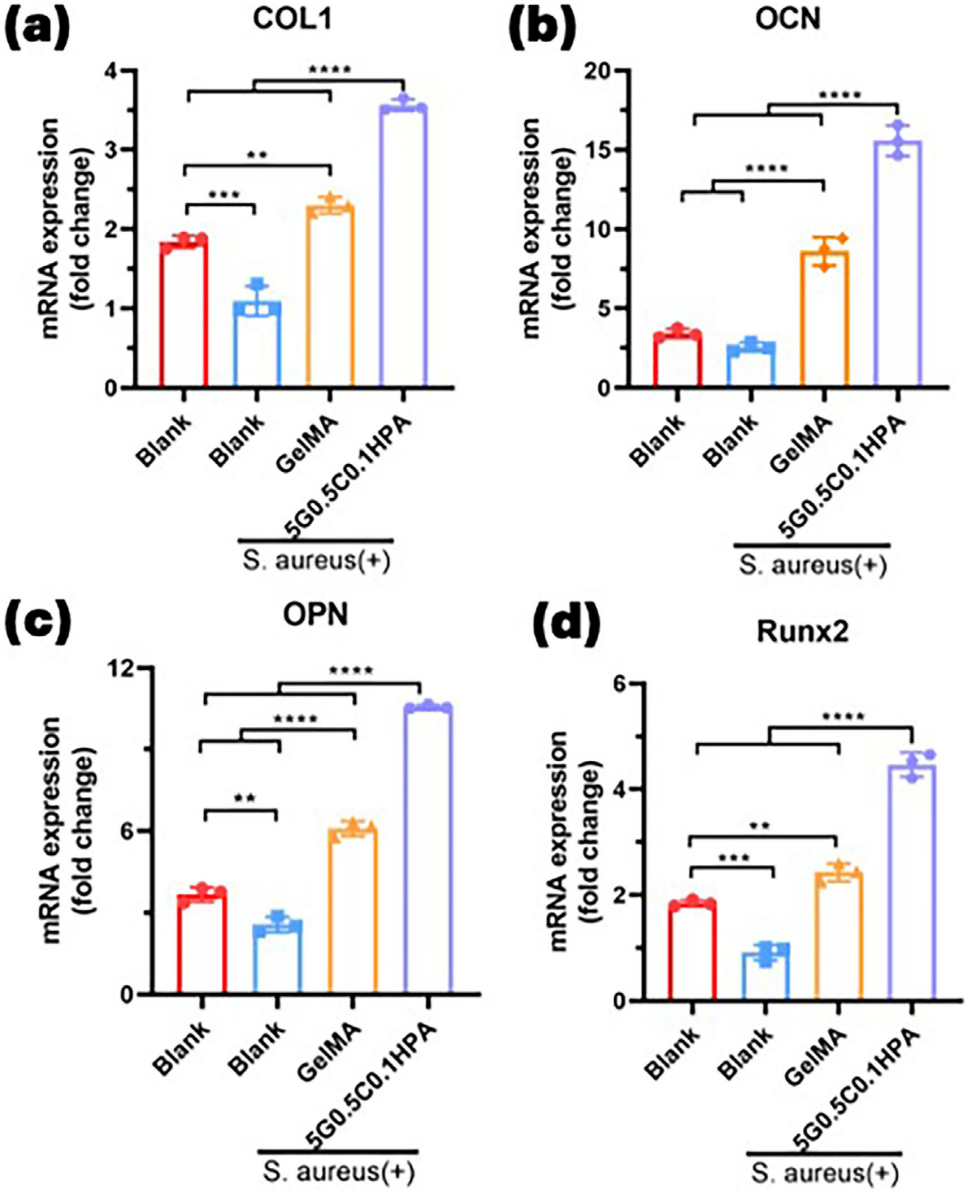
Relative mRNA expression levels of (a) COL1 (type I collagen), (b) OCN (osteocalcin), (c) OPN (osteopontin), and (d) RUNX2 (runt‐related transcription factor 2) were measured after 2 month of treatment. Data are presented as mean ± SD; statistical significance: *p* < 0.05 (*), *p* < 0.01 (**), *p* < 0.001 (***), *p* < 0.0001 (****).

Quantitative RT‐PCR analysis revealed that this composite significantly upregulated the expression of key osteogenic markers, including COL1, RUNX2, OPN, and OCN (Figure 8a–d), surpassing both the GeIMA and infected blank controls. The higher levels of RUNX2 and COL1 suggest that this material helps trigger early osteoblast differentiation, while the strong expression of OPN and OCN indicates good progress toward bone mineralization and matrix formation. Even under bacterial condition, this injectable G‐C‐HPA hydrogel showed strong performance in supporting bone formation and fighting off infection, demonstrating its dual function.

**FIGURE 9:**
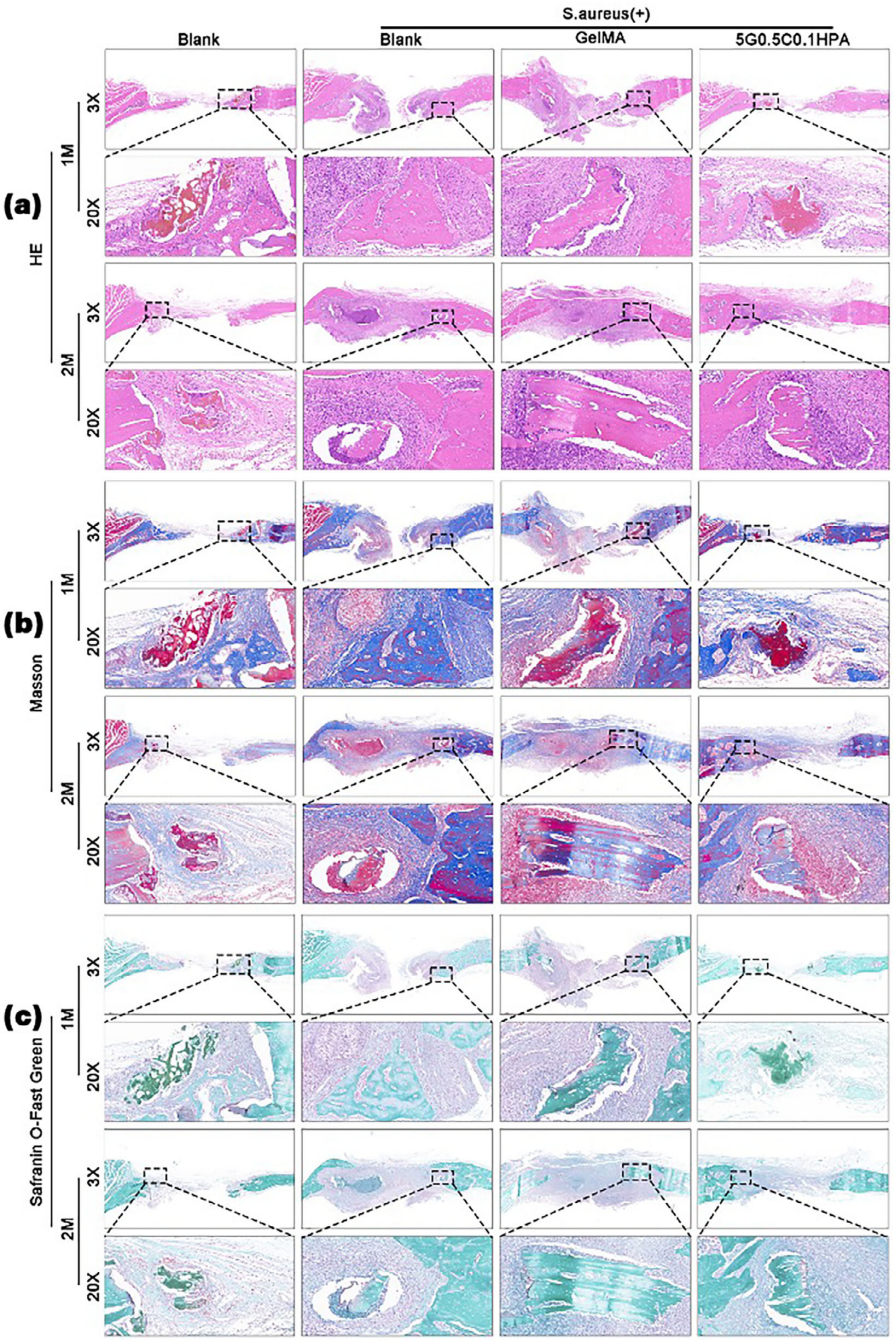
Pathology study of the skull defect model after 1 or 2 months of treatment. (a) H&E staining. (b) Masson staining. (c) Safrainin O‐Fast Green staining.

From H&E staining, after 1 month treatment, both negative blank group and G‐C‐HPA hydrogel group had a sign of new bone tissue formation (Figure 9a). In contrast, *SA* blank group and *SA* GelMA group had many inflammation cells. After 2 months of treatment, only G‐C‐HPA group showed a large area of mature new bone tissue, while the rest groups still had different levels of cavities. The conclusion is supported by the Masson staining result. In Masson staining, the natural healing group (negative blank group) only had very faint staining resulted from slow and weak bone formation (Figure 9b). In contrast, after the first month of treatment, G‐C‐HPA group started to show mature bone tissue, indicated by concentrated red staining. After 2 months of treatment, G‐C‐HPA group had a large area of well‐distributed bone (blue) filled with bone tissue (red). Safrainin O‐Fast Green is an indicator of cartilage soft tissue formation, where red shows cartilage soft tissue and blue/green shows bone tissue (Figure 9c). It was observed that G‐C‐HPA group had more contribution to forming bone tissue instead of cartilage tissue at 1 month of treatment. In the second month, the cartilage tissue significantly filled the bone tissue, forming a mature regenerated bone. There is no red staining observed for G‐C‐HPA group, showing no formation of cartilage soft tissue. Overall, upon pathology study of the in vivo skull defect model, G‐C‐HPA group had a superior healing performance from aspects of volume, maturation, and disinfection. With the successful skull defect healing model, it is promising that this injectable G‐C‐HPA hydrogel can be applied for other bone defect treatments. The injectability and high biocompatibility allow many applications on the non‐invasive surgery operation.

## 3 Materials and Methodology

### 3.1 Material

Methacrylic anhydride (MA), tetramethylethylenediamine (TEMED), ammonium persulfate (APS), N,N’‐Methylenebis(acrylamide) (MBA), dopamine hydrochloride (DA), and silver nitrate (AgNO_3_) were purchased from Sigma–Aldrich (MO, USA). Hydroxyapatite (Ca_5_(PO_4_)_3_OH) was purchased from Sima‐lab (China). Chitosan (< 200 mPa.s) and Acrylamide were purchased from Aladdin (Shanghai, China). Gelatin type A was purchased from DM Reagent (Tianjin, China). The Obtained chemicals were used in this project with no further purifications.

### 3.2 Synthesis

#### 3.2.1 Synthesis of Methacrylated Chitosan (ChiMA)

The method of synthesizing ChiMA followed a previously published method [80]. 3% (w/v) chitosan was first dissolved in 3% (w/v) acetic acid solution at room temperature for 24 h with gentle stirring. After chitosan powder was completely dissolved, methacrylic anhydride (MA) was added to the chitosan solution at 3.5:1 (chitosan to MA) w/v ratio. The mixture was stirred @ 300 rpm continuously for 3 h at room temperature. A homogeneous white opaque solution should be obtained. The mixture was then transferred into dialysis membrane (8–14 kDa) and submerged in deionized (DI) water for at least 4 days to remove the unreacted residuals under room temperature. The final obtained clear solution was lyophilized and stored at −20°C for further use.

#### 3.2.2 Synthesis of Gelatin Methacryloyl (GelMA)

Dissolve gelatin in DI water at a 10% (w/v) concentration under 50°C @500 rpm for at least 1 h until completely dissolved with no solid residues. MA was added into the gelatin solution dropwise at 1:1 (gelatin to MA) w/v ratio under 50°C and continuously stirred @ 500 rpm for 3 h until the solution became visually homogeneous. The solution was then transferred into a dialysis membrane (8–14 kDa) and immersed in deionized water (50°C) for at least 5 days to remove the unreacted residues. Next, the solution went through centrifugation @ 3000 rpm for 10 min to remove the unreacted solid residues. Finally, the clear solution was lyophilized and stored at −20°C for future use.

#### 3.2.3 Synthesis of HA‐PDA‐AgNP Powder (HPA)

0.2% (w/v) dopamine chloride was self‐polymerized in Tris Buffer for 30 min in an ambient environment to form poly‐dopamine (PDA). For 10 mL of PDA, add 100 mg of hydroxyapatite (HA) into the polymerized PDA and gently stirred under room temperature overnight (at least 8 h). The PDA grafted HA suspension was then washed with DI water 3 times and lyophilized subsequently. Obtained HA‐PDA powder was then added into 10 mL of 1% (w/v) AgNO_3_ solution and stirred under room temperature for another night. The Ag‐grafted HA‐PDA suspension went through the same wash‐lyophilization process, and eventually, the black HA‐PDA‐Ag powder was obtained. HA‐PDA‐Ag powder was denoted as HPA in final products such as 0.5Chi10Gel0.1HPA.

#### 3.2.4 Synthesis of Hydrogel

The synthesis of injectable HPA‐embedded hydrogel was facile. 1% (w/v) ChiMA solution needed to be prepared at least 1 day before hydrogel synthesis by simply dissolving lyophilized ChiMA in DI water in RT without stirring to avoid bubbles. Clear ChiMA solution would be obtained after 24 h of dissolution. 20% (w/v) of ammonium persulfate (APS) solution was prepared fresh daily in DI water. To prepare 1 mL of 0.5Chi10Gel0.1HPA hydrogel (0.5% ChiMA, 10% GelMA and 0.1% HPA powder), dissolve 100 mg of dry GelMA foam in 500 µl of DI water in 50°C water bath for 30 min. After GelMA was completely dissolved, add 1 mg of HPA powder. The mixture was subjected to sonication for 10 min until the black powder was averagely dispersed in the solution. Then, add 500 µl of prepared 1% ChiMA and 20 µl of 20% APS solution. Immediately, the mixture was vortexed and let stand for polymerization.

The control group of G‐C‐TEMED was synthesized by mixing prepared GelMA, ChiMA, and APS solution with the same concentration mentioned above, and then adding 2 uL of TEMED for every 1 mL of hydrogel. The control group of UV light assisted polymerization was synthesized by preparing 0.2% (w/v) I2959 solution, then mixing the solution with designed concentration of GelMA and ChiMA to have a final concentration of I2959 to be 0.1% in the hydrogel precursor. The vortexed precursor was presented under UV light for 2 min for polymerization.

### 3.3 Characterization

The functional groups were inspected through Fourier transform infrared (FTIR) spectra (Nicolet is 20, USA) in an ambient environment. The scanning electron microscope (SEM) was adopted to study the micro‐morphological structure of compound hydrogel (FEI Nova NanoSEM 450). The lyophilized hydrogel samples were sputtered with gold prior to SEM photography. Energy dispersive X‐ray spectroscopy element mapping (EDS) was also carried out on the same device at a voltage of 10.0 kV. The diameter of powders was analyzed at room temperature on a Zetasizer Nano series (Malvern Instruments Ltd., Malvern, UK). All ^1^H NMR experiments were conducted on a Bruker Avance 300 MHz NMR spectrometer, and the relaxation delay (d1) was set as 2 s. The samples were dissolved in D_2_O at the concentration of 10 mg mL^−1^. A quantity mount of pyrrole was added into the NMR solution of GelMA as external standard for quantification of the amount of methacrylate groups grafted onto gelatin or elastin. All NMR data were provided in the Supporting Information. To determine the concentrations of Ag ions, ICP‐MS (Agilent 8900 ICP‐MS/MS) was reported. Releasing Ag ions samples were prepared by immersing the G‐C‐HPA hydrogel in water on 37° heated plate for 1, 3, 5, and 7 days and then preserved in 1% HNO_3_ until analysis.

### 3.4 Rheology

All rheology experiments, including time sweep, frequency sweep, temperature sweep and viscosity sweep, were conducted and processed with TA Discovery Hybrid Rheometer using an 8 mm Peltier plate. The hydrogels were positioned on the parallel plate. The samples were measured in oscillation‐frequency experiments using parameters of frequency ranging from 0.1 to 10 Hz and the constant strain of 0.1% at 37°C if not mentioned elsewhere.

### 3.5 Uniaxial Compressive Testing

Hydrogel samples were synthesized in a cylinder container and shaped as 8 mm diameter and 6 mm height. The prepared samples were tested in a compressive mode (MTS Criterion 43 Model, loading cell 1000N) at a crosshead speed of 1.0 mm min^−1^. The compression threshold was 80% of the compression strain to avoid damaging the load cell. The young's modulus was estimated by the slope of the stress σ and strain ε where:

$$E=\frac{\sigma }{\varepsilon }$$

### 3.6 Swelling Ratio and Porosity

The weights of each dry lyophilized sample were measured in advance as *Wd*. Then, lyophilized samples were incubated in phosphate‐buffered saline (PBS) at 37°C for 24 h to reach an equilibrium weight. After 24 h, the swollen samples were carefully taken out from the container and wiped with filter paper to remove the excess liquid on the surface. The swollen weight was recorded as *Ws*.

The swelling ratio (SR) was then calculated as:

$$SR\%=\frac{Ws-Wd}{Wd}\times 100\%$$

The liquid displacement method was employed to measure the porosity of water absorbing materials [81]. The lyophilized dry sample was first placed in a 1 mL viral, deionized water was slowly added to the viral until it reached 1 mL (including solid and liquid). The added volume was documented as V_0_. Let the sample absorb water of 37°C. After 24 h, the wet sample was carefully taken out. The volume of water that was left in the viral was documented as V_1_. The porosity (P) of each sample was then calculated as:

$$P\%=\frac{\left(1-V_{1}\right)-\left(1-V_{0}\right)}{\left(1-V_{0}\right)}\times 100\%$$

### 3.7 Degradation of the Hydrogels

The degradation of hydrogels was measured by immersing the hydrogel samples in PBS buffer (pH = 7.4, 37°C). The weights of hydrogel samples were measured on day 0, 1, 3, and 7. The documented weight change was used to evaluate the degradation degree of samples.

### 3.8 Antibacterial Activity In Vitro

The antibacterial activity was demonstrated by an inhibitory zone method using *E. coli* and *S. aureus* bacteria. Samples were prepared into groups of GelMA, ChiMA, and G‐C‐HPA composite hydrogel. LB broth was prepared using DI water and then subjected to autoclaving at 121°C for 15 min. The chosen bacteria were cultured at 37°C @ 250 rpm shaking speed overnight. The bacteria strain was then spread on Tryptic Soy Agar plate. The samples were then sliced and placed on the plate. The antibacterial activity was evaluated by how the sample materials can inhibit the bacteria growth on the culture plate.

### 3.9 Cytotoxicity Test

The cell viability was tested through a CCK8 kit. Briefly, L929 cells were cultured in a regular medium for 24 h in a 96‐well plate with 4 × 10^3^ cells in each well. On the side, sample materials were also cultured in a cell culture medium to prepare the sample medium. Once the sample medium was ready, the original L929 cell medium was replaced with 90 µL sample medium and 10 µL of CCK8 solution (on day 1 and day 3). The cell vitality was evaluated through the absorbance value (450 nm, *n* = 3).

A Live/Dead assay was also adopted to demonstrate the cytotoxicity. The Live/Dead Viability kit was prepared in PBS buffer to achieve a final concentration of calcein AM of 1 µL mL^−1^ and propidium iodide PI of 3 µL mL^−1^. The L929 cells were cultured in a sample material medium. On Day 1 and Day 3, the cultured medium was subjected to be treated in a probe solution at 37°C for 30 min. The fluorescence photography was collected by an inverted fluorescence microscope. The green fluorescence indicated live bacteria cells which were stained with calcein AM; in contrast, red fluorescence represented dead cells stained with propidium iodide. The vitality level of L929 cells was demonstrated by living and dead cells counts using ImageJ.

### 3.10 Bone Defect Healing In Vivo

All in vivo experiments were performed with approval from the Animal Research Committee of Nanchang University of China. To obtain a rat model of skull defect infection, 16‐week‐old female SD rats underwent critical‐size skull defect surgery. *S. aureus* (1 × 10^9^ CFU mL^−1^) was cultured in the medullary cavity of the bone defect after surgery. Briefly, 32 rats weighted 350 to 400 g were randomly separated into four groups. All rats were anesthetized by intraperitoneal injection of phenobarbital (35 mL kg^−1^) and fixed on the operating table (2022042004A, Zhongke Huida Technology). Next, a critical size defect with a diameter of 5 mm was created using an electric drill, and different hydrogels were injected into the defect area: 1) Negative blank group (*n* = 8, without bacteria), 2) *SA* blank group (*n* = 8, applied with the bacterial culture after surgery), 3) *SA* GelMA group (*n* = 8, GelMA hydrogel added after bacterial culture and surgery), and 4) 5G0.5C0.1HPA (*n* = 8, hydrogel precursor added after bacterial culture and surgery). The wound was then sutured by layers.

On the 3rd day after initial surgery, *SA* was washed from the bone marrow cavity with PBS and cultured on agar plates. The in vivo antibacterial activity was observed through counting.

The pathology was further analyzed through H&E, Masson and Safranin O‐Fast Green staining. The specimens were collected from the experimental rat models mentioned above. The specimens were fixed with 4% paraformaldehyde solution, dehydrated in gradient ethanol, embedded in paraffin, and finally sectioned for staining.

## 4 Conclusions

In this report, we described an innovative bone filling gel, which is a highly bio‐compatible hydrogel precursor that could self‐polymerize in vivo within 10 min. This precursor was constituted of GelMA, ChiMA, HA, PDA and AgNPs. HA, PDA and AgNPs were synthesized and acted as an anti‐bacterial agent and catalyst. HA had rough surface that could increase the surface‐volume ratio of AgNPs, which significantly accelerated the self‐polymerization without any external stimuli. With such combination, this precursor had low viscosity that endowed smooth injection through very thin syringe that could reach internal organs through non‐invasive surgery procedures.

Upon examination, the precursor was proved to have high cell affinity, low cytotoxicity, and excellent anti‐bacterial activity with HPA concentration at 0.1%. After the concentration of 5G0.5C0.1HPA was determined, the precursor was applied on an in vivo infected skull animal model. With the application of our G‐C‐HPA precursor, the bacteria were eliminated efficiently without further corruption on adjacent bone tissues. The skull defect could regenerate roughly 50% of its original volume after 2 months of G‐C‐HPA treatment. The regenerated bone tissues were examined to be more mature than the contrast groups.

As a result, the G‐C‐HPA injectable precursor is demonstrated as a solution of commonly reported bone cement failures with huge potential in non‐invasive bone surgery treatment.

## Conflicts of Interest

The authors declare no conflicts of interest.

## Supporting information




**Supporting File**: mabi70084‐sup‐0001‐SuppMat.doc.

## Data Availability

The data that support the findings of this study are available from the corresponding author upon reasonable request.
