# Supplementary material for: Injectable Self‐Polymerizing Hydrogel as Bone Filler for Bone Defect Treatment
Source: Macromol Biosci. 2025 Oct 13;25(12):e00281. doi: 10.1002/mabi.202500281 (PMC12704240; doi:10.1002/mabi.202500281)
Supplement: Supplementary file 1 — Supporting File: mabi70084‐sup‐0001‐SuppMat.doc. [file MABI-25-e00281-s001.doc]

# Supporting Information

#### 1. Methacrylic Anhydride (MA) Degree for GelMA and ChiMA


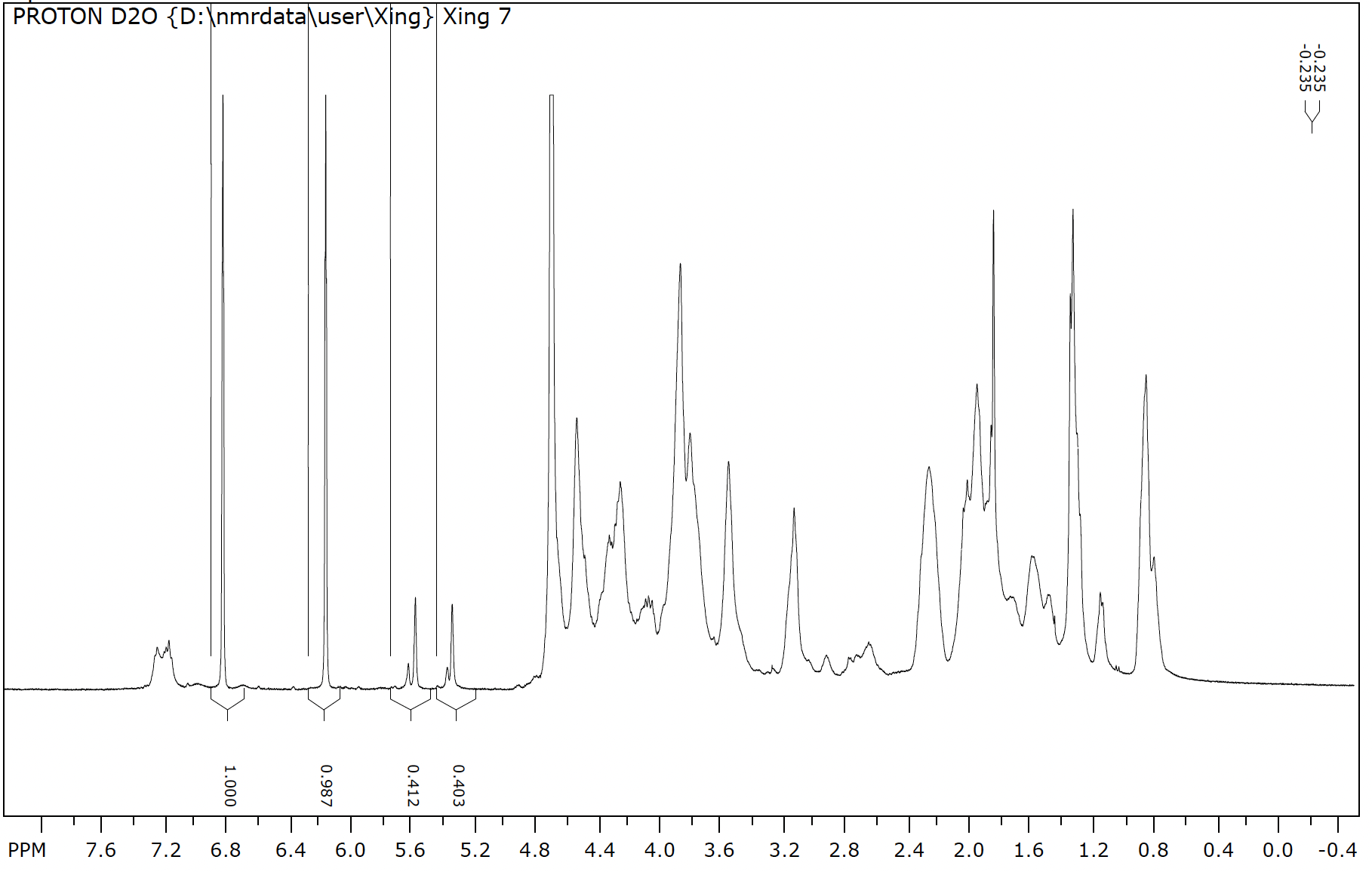


**Figure S1** ^1^H NMR spectrum of 12 mg GelMA doped with 0.5mg pyrrole as external standard.

The calculation for MA degree is in reference of previous reports [1, 2]. The peaks at 5.3 ppm and 5.6 ppm were assigned to methacrylate. While the peaks at 6.3 ppm and 6.8 ppm were assigned to external standard pyrrole. The calculated mole of MA in sample was 0.255 mmol per 1 g of GelMA according to the equation below.


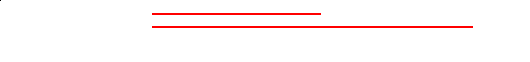


The calculated MA degree was ~ 85%.


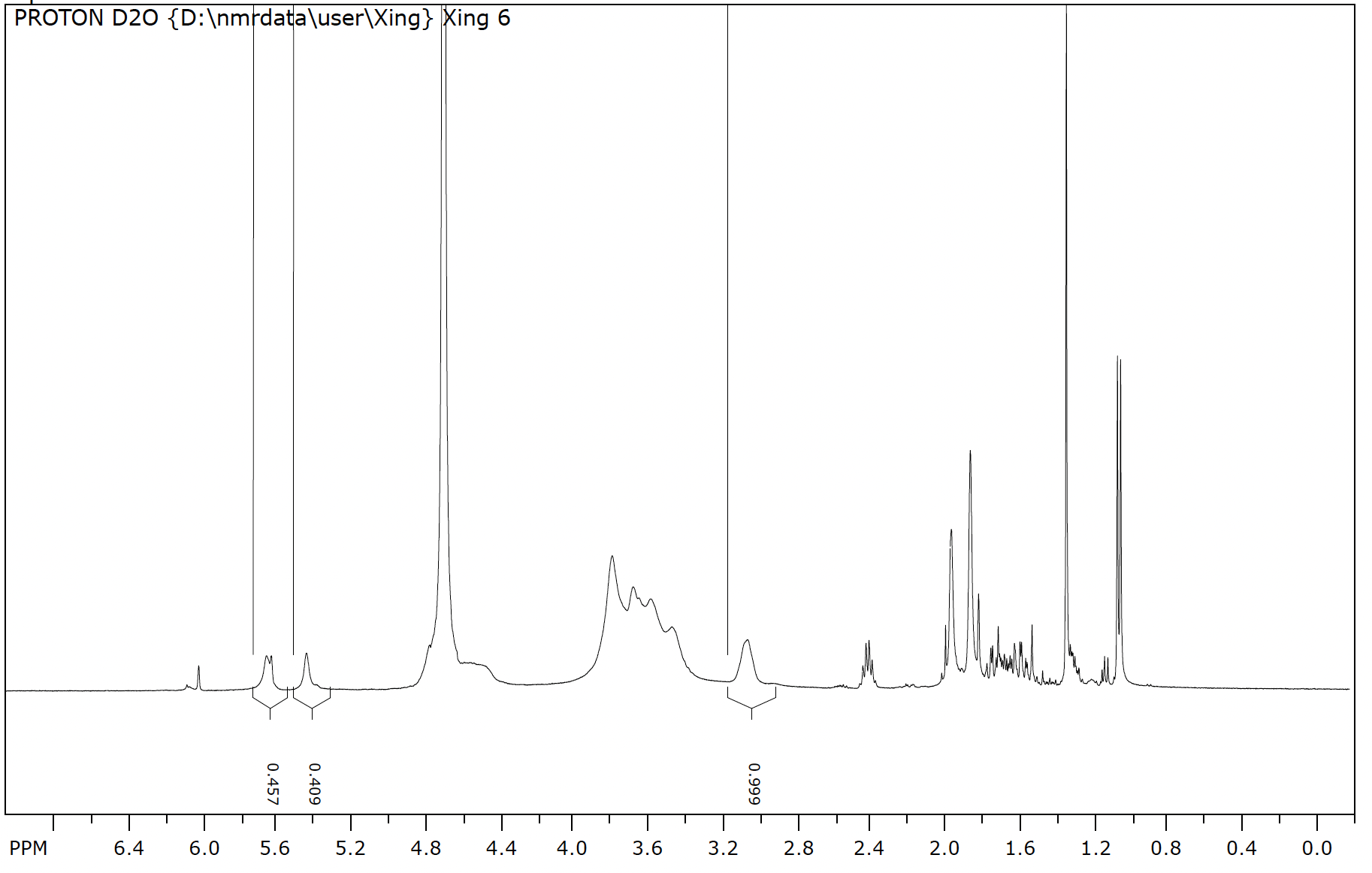


**Figure S2** ^1^H NMR spectrum of ChiMA dissolved in D_2_O with HCl to accelerate the dissolving process.

The calculation of MA degree for ChiMA was in reference to previous report [3]. The peaks at 5.4 ppm and 5.6 ppm were assigned to MA and peak at 3.1 was assigned to proton on chitosan backbone. The MA degree is calculated as:


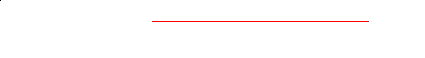


The calculated MA degree was ~ 43.3%.

#### 2. Ag wt% calculation

| Element | Weight % | Atomic % | Net Int. | Error % |
| --- | --- | --- | --- | --- |
| C K | 43.03 | 51.03 | 482.32 | 6.16 |
| N K | 19.66 | 19.99 | 75.36 | 11.38 |
| O K | 28.94 | 25.77 | 260.16 | 9.07 |
| AlK | 1.02 | 0.54 | 22.92 | 7.52 |
| P K | 0.73 | 0.33 | 12.07 | 13.22 |
| S K | 3.89 | 1.73 | 60.17 | 4.28 |
|
| AgL | 1.59 | 0.21 | 8.53 | 23.48 |
| CaK | 1.16 | 0.41 | 7.73 | 17.00 |

**Table S1** EDS report of lyophilized 5G0.5C0.1HPA sample.

A total of 1 mg of HPA (includes hydroxyapatite and AgNPs) was added in 1 mL of hydrogel precursor to form 5G0.5C0.1HPA hydrogel.


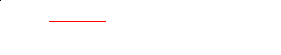


Where

W_Ag_​ = Actual mass of AgNPs in the total measured HPA powder (mg).

wt% Ag and wt% Ca = Measured weight percentage of Ag and Ca from EDS (%).

1 mg = Total measured mass of HPA powder in 1 ml of hydrogel (including both hydroxyapatite and AgNPs).

39.9% = Theoretical mass fraction of Ca in pure hydroxyapatite (Ca_5_(PO₄)_3_OH).

From the weight % between Ag and Ca in the EDS report, we can calculate that per 1 ml of hydrogel sample has ~ 353 µg of AgNPs.

#### 3. Gradual Release of Ag Ions


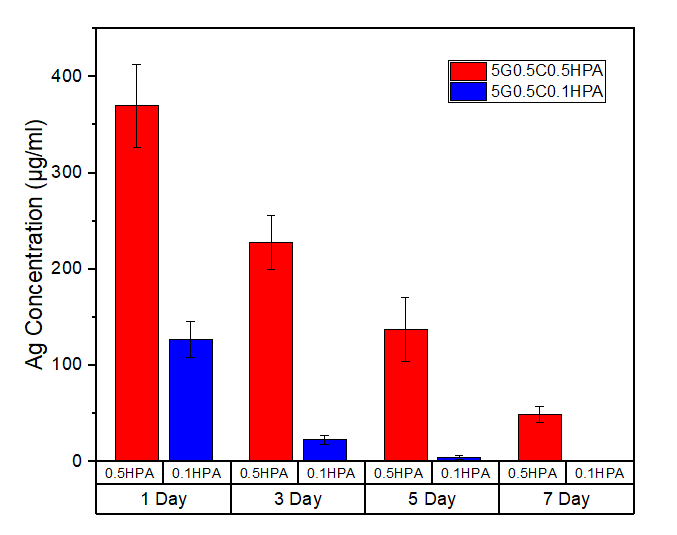


**Figure S3** The gradual release of Ag ions from the sample hydrogels.

The concentration samples were collected by placing a 1 ml hydrogel sample in 5 ml of water on top of 37 ° heated plate for the periods listed in the graph. Then the water was collected and then subject to the concentration profile. The data listed here was multiplied by 5 to assume a 1 ml hydrogel sample in 1 ml water situation for easier comparison.

1. Zhu, M., et al., *Gelatin methacryloyl and its hydrogels with an exceptional degree of controllability and batch-to-batch consistency.* Sci Rep, 2019. **9**(1): p. 6863.

2. Liu, Y., et al., *Highly Flexible and Resilient Elastin Hybrid Cryogels with Shape Memory, Injectability, Conductivity, and Magnetic Responsive Properties.* Adv Mater, 2016. **28**(35): p. 7758-67.

3. Cao, Y., et al., *Designing siRNA/chitosan-methacrylate complex nanolipogel for prolonged gene silencing effects.* Sci Rep, 2022. **12**(1): p. 3527.
